# Identification of Neurocan and Phosphacan as Early Biomarkers for Open Neural Tube Defects

**DOI:** 10.3390/cells12071084

**Published:** 2023-04-04

**Authors:** Karolina Janik, George M. Smith, Barbara Krynska

**Affiliations:** 1 Shriners Hospitals Pediatric Research Center, Lewis Katz School of Medicine, Temple University, 3500 North Broad Street, Philadelphia, PA 19140, USA; 2 Department of Neural Sciences, Lewis Katz School of Medicine, Temple University, 3500 North Broad Street, Philadelphia, PA 19140, USA

**Keywords:** neural tube defect, spinal cord injury, myelomeningocele, amniotic fluid, neurocan, phosphacan

## Abstract

Open neural tube defects (NTDs) such as myelomeningocele (MMC) are debilitating and the most common congenital defects of the central nervous system. Despite their apparent clinical importance, the existing early prenatal diagnostic options for these defects remain limited. Using a well-accepted retinoic-acid-induced model of MMC established in fetal rats, we discovered that neurocan and phosphacan, the secreted chondroitin sulfate proteoglycans of the developing nervous system, are released into the amniotic fluid (AF) of fetal rats displaying spinal cord defects. In contrast to normal controls, elevated AF levels of neurocan and phosphacan were detected in MMC fetuses early in gestation and continued to increase during MMC progression, reaching the highest level in near-term fetuses. The molecular forms of neurocan and phosphacan identified in the AF of MMC fetuses and those found in MMC spinal cords were qualitatively similar. In summary, this is the first report demonstrating the presence of neurocan and phosphacan in the AF of MMC fetuses. The identification of elevated levels of neurocan and phosphacan in the AF of MMC fetuses provides two prospective biomarkers with the potential for early prenatal diagnosis of open NTDs.

## 1. Introduction

Myelomeningocele (MMC), commonly known as open spina bifida, is a severely disabling open neural tube defect (NTD) that affects the developing spinal cord. It occurs in approximately 3 per 10,000 live births per year in the United States and is clinically very important [1,2,3]. The presentation is commonly in the lumbosacral region, and the defect is characterized by protrusion of the malformed spinal cord and meninges through a pathological opening in the overlying vertebrae and skin, leaving the spinal cord exposed to the intrauterine environment [4]. The underlying defect leads to prenatal injury to the exposed spinal cord and a spectrum of associated abnormalities resulting in lifelong disability, including leg paralysis, sensory loss, bowel and bladder dysfunctions, skeletal deformations, Arnold-Chiari type II malformation, hindbrain herniation, and development of hydrocephalus [5,6,7,8,9]. Affected individuals have profoundly diminished quality of life, often requiring lifelong support and institutional care [10,11].

The progressive nature of MMC over the course of gestation with lifelong clinical impact points to the importance of early prenatal diagnosis, which provides an opportunity for timely management decisions. However, diagnosis of open NTDs is challenging, particularly in the early stages. Currently, measurement of alpha fetoprotein (AFP) levels and/or fetal imaging are typically used for prenatal diagnosis of open NTDs. While elevated levels of AFP in maternal serum and amniotic fluid (AF) are used for screening and diagnosis of open NTDs, respectively, the specificity and sensitivity of open NTD detection by AFP is limited [12,13,14,15]. Therefore, there is a need for the development of biomarkers enabling early and accurate prenatal diagnosis of open NTDs. 

The chondroitin sulfate proteoglycans (CSPGs) neurocan and phosphacan are abundant extracellular matrix (ECM) components of the developing central nervous system (CNS) synthesized by neural stem cells, astrocytes, and neurons [16,17]. With the occurrence of open NTD, we hypothesized that these highly soluble forms of ECM proteins [18,19] would be released from the site of the spinal cord defect into the surrounding AF, resulting in increased levels of neurocan and phosphacan in the AF of affected fetuses. Therefore, using a well-established retinoic acid (RA)-induced fetal rat model of MMC [20,21], the objective of this study was to determine whether AF levels of neurocan and/or phosphacan are elevated in affected fetuses compared to normal age-matched controls at various points of gestation, particularly in early development, and whether elevated levels of neurocan and phosphacan may constitute potential biomarkers for open NTDs. The use of an RA-induced fetal rat model of MMC, which results from the incomplete closure of the neural tube, allows for a systemic analysis of samples and age-matched controls at various points of gestation. In parallel, we analyzed the expression of neurocan and phosphacan in MMC spinal cords and determined the solubility and in vitro release of neurocan and phosphacan from the MMC spinal cord tissue into the AF. 

## 2. Materials and Methods

### 2.1. Retinoic-Acid-Induced Animal Model of MMC 

Time-dated pregnant Sprague–Dawley rats (Charles River Laboratories, Wilmington, MA, USA) were placed on a standard dark:light schedule. An MMC defect was induced in fetuses of time-dated pregnant rats by gavage of a single dose of 50 mg/kg of all-trans retinoic acid (RA) (Sigma Aldrich, Saint Louis, MO, USA) dissolved in olive oil on embryonic day 10 (E10), as described previously [21,22]. Normal control dams were gavage-fed with olive oil. Dams were euthanized using chamber-inhaled CO_2_, and the total number of fetuses was determined following the midline laparotomy and exposure of the uterus. AF samples from fetuses with MMC defect after RA exposure (*n* = 91), no MMC defect after all-trans RA exposure (*n* = 8), and normal fetuses (*n* = 97) were collected at E14, E16, E18, and E21 (term = E22) using aseptic techniques. After collection of AF samples, the uterus and gestational membranes were removed, and each fetus was examined for the presence of lumbosacral MMC defect. An incidence of isolated MMC defects was observed in 92% (91/99) of fetuses. After harvesting, fetuses were collected and euthanized according to standard procedures. 

### 2.2. AF Preparation

Immediately after harvesting, AF samples collected from fetuses at E14, E16, E18, and E21 were centrifuged at 4000 rpm for 10 min to remove cells and debris, then stored at −80°C until further analysis. Prior to Western blotting, equal volumes of AF from 3 randomly selected MMC or normal AF samples were combined and digested with 0.03 international units (IU) of chondroitinase ABC (ChABC; Sigma Aldrich, Saint Louis, MO, USA) in 0.1 M Tris-HCl buffer (pH = 8.0) containing 0.03 M sodium acetate and protease inhibitor cocktail (1:100; Sigma Aldrich, Saint Louis, MO, USA) for 3 h at 37 °C with gentle shaking (18). Enzymatic reaction was terminated by adding 4× Laemmli sample buffer (Bio-Rad Laboratories, Hercules, CA, USA) with 10% β-mercaptoethanol (Sigma Aldrich, Saint Louis, MO, USA). AF samples were subsequently subjected to gel electrophoresis and analyzed by Western blotting as described below. 

### 2.3. Protein Isolation

#### 2.3.1. Total Spinal Cord Protein Extracts

Lumbar spinal cords were isolated from E21 MMC fetuses collected as described above. Immediately after harvesting, lumbar spinal cord samples from 6–7 randomly selected fetuses were pooled together and collected onto 50 mM Tris-HCl buffer with 0.5% Triton X-100 (Thermo Fisher Scientific, Waltham, MA, USA) and protease inhibitor cocktail. Samples were subsequently homogenized, rotated at 4 °C for 45 min, and centrifuged at 14,000× *g* for 30 min at 4 °C. Protein concentration in spinal cord extracts was measured using a colorimetric detection and quantification kit and a Pierce^TM^ BCA protein assay kit (Thermo Scientific, Rockford, IL, USA), and samples were stored at −80 °C until further analysis. Prior to gel electrophoresis, spinal cord protein samples (10 µg) were digested with ChABC as described above for AF preparation, subjected to gel electrophoresis, and analyzed by Western blotting. Total protein extracts of MMC spinal cords served as positive controls for Western blotting analyses of the AF.

#### 2.3.2. Sequential Spinal Cord Protein Extracts 

Lumbar spinal cords were isolated from MMC fetuses collected at E14, E16, E18, or E21 as described above. Immediately after harvesting, lumbar spinal cords from 6–7 randomly selected fetuses per age group were pooled together and collected onto 50 mM Tris-HCl buffer with protease inhibitor cocktail. Samples were homogenized, rotated at 4 °C for 45 min, and centrifuged at 14,000× *g* for 30 min at 4 °C (1st extract). The resultant pellets were lysed with buffer composed of 50 mM Tris-HCl with 0.5% Triton X-100 and protease inhibitors, rotated at 4 °C for 45 min, and centrifuged at 14,000× *g* for 30 min at 4 °C (2nd extract). Protein concentration was measured using a colorimetric detection and quantification kit and a Pierce^TM^ BCA protein assay kit, and samples were stored at −80 °C until further analysis. Prior to gel electrophoresis, 1st and 2nd protein extracts (10 µg each) were digested with ChABC and subjected to Western blotting analyses as described below. All analyses were performed using spinal cord extracts prepared from at least two independent sets of spinal cord tissue samples per age group. 

#### 2.3.3. MMC Spinal Cord Protein Extracts in Normal AF

Lumbar spinal cords were isolated from E21 MMC fetuses collected as described above. To determine the solubility of neurocan and phosphacan into the AF, lumbar spinal cords from 6–7 randomly selected fetuses were pooled together and collected in the AF of normal age-matched fetuses. Samples were rotated for 45 min at 4 °C and centrifuged at 14,000× *g* for 30 min. Prior to gel electrophoresis, equal volumes of spinal cord extracts were digested with ChABC and subjected to Western blotting analyses as described below. All analyses were performed using spinal cord extracts prepared from at least two independent sets of MMC spinal cord tissue samples and AF collected from normal age-matched fetuses. 

### 2.4. Western Blotting

Amniotic fluid or spinal cord samples were resolved on 8% sodium dodecyl sulfate-polyacrylamide gel (SDS-PAGE) and transferred onto 0.22 µM nitrocellulose membranes (Li-Cor Biosciences, Lincoln, NE, USA). After transfer, blots were blocked for non-specific binding with 5% blotting-grade blocker (Bio-Rad Laboratories, Hercules, CA, USA) in TBS and incubated with one of the following antibodies: 1F6 neurocan mouse monoclonal antibody (1:500; Developmental Studies Hybridoma Bank—DSHB, Iowa City, IA, USA), 650.24 neurocan mouse monoclonal antibody (1:1000; Santa Cruz Biotechnology, Dallas, TX, USA), or 3F8 phosphacan mouse monoclonal antibody (1:250; DSHB, Iowa City, IA, USA). The sizes of the detected proteins were estimated using Precision Plus Protein™ all-blue prestained protein standards (Bio-Rad Laboratories, Hercules, CA, USA). Blots were incubated with goat anti-mouse antibody conjugated to IRDye^®^ 680RD dye (LI-COR Biosciences, Lincoln, NE, USA), and signals were detected using Odyssey CLx Imaging System (LI-COR Biosciences, Lincoln, NE, USA). Image Studio Ver. 3.1. (LI-COR Biosciences, Lincoln, NE, USA) was used to quantify the signal intensity and presented as arbitrary fluorescence units (AFU). 

### 2.5. Histology and Immunofluorescence 

Fetal rats were collected as described above, then fixed in 10% neutral buffer formalin (Thermo Fisher Scientific, Waltham, MA, USA) at 4 °C. After fixation, samples were equilibrated in 15% followed by 30% sucrose in PBS at 4 °C before being embedding in OCT (Sakura Finetek, Torrance, CA, USA) and frozen. Serial 15 µm coronal sections were obtained through the center of the MMC defect and mounted on charged glass slides (Superfrost Plus, Fisher Scientific, Pittsburgh, PA, USA). For histological analyses, sections were stained with H&E (Sigma Aldrich, Saint Louis, MO, USA) according to the manufacturer’s protocol, mounted with VectaMount^®^ permanent mounting medium (Vector Laboratories; Burlingame, CA, USA), cover-slipped, and visualized using a bright-field microscope (Eclipse 80i; Nikon). Sections assigned for immunofluorescence were blocked with 5% donkey serum (Sigma Aldrich, Saint Louis, MO, USA) in 1X bovine serum albumin (BSA; Sigma Aldrich, Saint Louis, MO, USA) solution with 0.3% Triton X-100 in PBS for 45 min at room temperature. Sections were then incubated with 650.24 (1:250) or 3F8 (1:150) antibodies and subsequently with donkey anti-mouse Alexa Fluor^®^ 555 secondary antibody (1:1000; Invitrogen, Rockford, IL, USA) and 4′6′-diamidini-2-phenylindole (DAPI; Sigma Aldrich, Saint Lois, USA) to visualize cell nuclei. Sections when then mounted with ProLong Gold antifade reagent (Invitrogen, Eugene, OR, USA) and cover-slipped. Photographs were taken using a Leica SP8 confocal microscope (Leica). All analyses were performed using cross sections obtained from the lumbar spinal cord region of three MMC fetuses collected at E21 by examining at least three sections from each fetus. 

### 2.6. RNAscope and Coimmunostaining

For RNAscope, sections were processed using an RNAscope^®^ V2 multiplex fluorescent reagent kit (Advanced Cell Diagnostics Inc.—ACD, Newark, CA, USA) and probes specifically targeting rat *Neurocan* (Rn-Ncan-C2; ACD, Newark, CA, USA) and rat *PTPRZ/Phosphacan* (Rn-Ptprz1-C3; ACD, Newark, CA, USA, which recognizes all alternatively spliced transcript variants) according to the manufacturer’s protocol. Cell nuclei were stained with DAPI. Sections were then mounted with ProLong Gold antifade reagent and cover-slipped. Photographs were taken using a Leica SP8 confocal microscope. All analyses were performed using cross sections obtained from the lumbar spinal cord region of three MMC fetuses collected at E14 and E21 by examining at least three sections from each fetus. 

RNAscope with coimmunostainings were carried out using an RNA protein codetection ancillary kit (ACD, Newark, CA, USA), mouse anti-MAP2 (MAB3418; 1:100; Millipore, Burlington, MA, USA), and rabbit anti-glutamine synthetase (ab73593; 1:200; Abcam, Boston, MA, USA) antibodies according to the manufacturer’s protocol. Cell nuclei were stained with DAPI. Sections when then mounted with ProLong Gold antifade reagent and cover-slipped. Photographs were taken using a Leica SP8 confocal microscope. All analyses were performed using cross sections obtained from the lumbar spinal cord region of three MMC fetuses collected at E21 by examining at least two sections from each fetus. 

### 2.7. Statistical Analysis 

Statistical analyses were conducted using GraphPad Prism 8.3.0 software (GraphPad Software, LLC, Boston, MA, USA). Data were analyzed using an unpaired t-test or ab ordinary one-way ANOVA with Tukey’s comparison test when applicable. All numerical data are presented as mean ± SD.

## 3. Results

### 3.1. Elevated Levels of Neurocan and Phosphacan in the AF of MMC Fetuses

**Figure 1:**
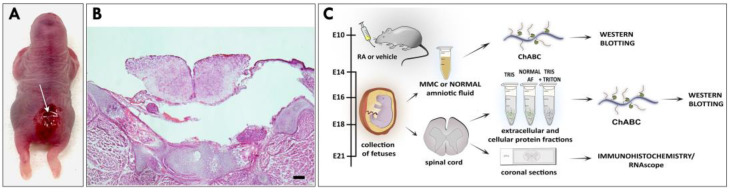
(**A**) Representative external view of RA-induced MMC defect in the lumbar region of a fetal rat at E21. Arrow indicates the beginning of the exposed spinal cord. (**B**) Representative H&E-stained cross section from the MMC defect in a fetal rat at E21 demonstrating a malformed spinal cord and failed development of overlying structures. Scale bar indicates 200 µm. (**C**) Overview of the experimental strategy.

**Figure 2:**
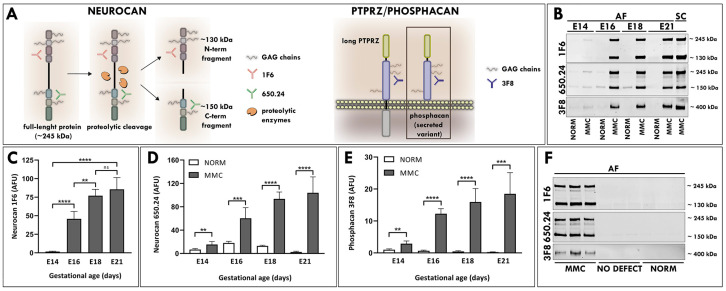
(**A**) Schematic representation of the domain organization of neurocan and its major proteolytic fragments, N-terminal fragment, and C-terminal fragment, as well as a long splice form of PTPRZ and its extracellular variant, phosphacan. Domains recognized by 1F6 and 650.24 and 3F8 antibodies are indicated. (**B**) Equal volumes of AF samples pooled from three randomly selected MMC fetuses or age-matched normal fetuses collected at E14, E16, E18, and E21 were digested with ChABC, loaded on the gel, and subjected to Western blotting analyses. A representative Western blot with 1F6 and 650.24 antibodies illustrates detection of full-length neurocan and the N-terminal fragment (245 kDa and 130 kDa) in the AF of MMC fetuses at all embryonic ages and full-length neurocan and the C-terminal fragment (245 kDa and 150 kDa). Analogous Western blotting analysis with the 3F8 antibody demonstrates detection of phosphacan (~400 kDa) in the AF of MMC fetuses at all embryonic ages. In the SC lane, MMC spinal cord lysates were loaded on the gel as positive controls. (**C**–**E**) Quantitative analyses of total neurocan and phosphacan proteins in the AF of MMC fetuses and the AF of age-matched normal controls. Graphs represent the change in arbitrary fluorescence units (AFU) at each time point. Data are presented as mean ± SD of 15 randomly selected AF samples per group., ** *p* < 0.01, *** *p* < 0.001, **** *p* < 0.0001. (**F**) Equal volumes of AF samples from randomly selected individual fetuses collected at E21 were digested with ChABC loaded on the gel and subjected to Western blotting analyses with 1F6, 650.24, and 3F8 antibodies. Representative Western blot illustrates detection of neurocan and phosphacan in all AF samples from individual E21 fetuses with MMC defect but very weak or no neurocan and phosphacan detection in littermates with no MMC defect after RA exposure and normal controls. Data represent six AF samples per group.

For this study, MMC was induced during neurulation by exposure to all-trans retinoic acid (RA) [21]. As previously reported, MMC fetuses displayed defects characterized by a pathological opening in the vertebral arch and the overlying skin, with the spinal cord exposed at the center of the lesion confined to the lumbosacral area of the fetus [20,21,23] (Figure 1A,B). AF samples collected from fetal rats with MMC defects and age-matched normal controls at four time points in gestation (E14, E16, E18 and E21) were treated with chondroitinase ABC (ChABC) to degrade polysaccharides and examined using Western blotting (Figure 1C). Following immunoblotting with the 1F6 antibody, which recognizes the N-terminal epitope of neurocan (Figure 2A), a faint band representing a full-length neurocan (245 kDa core protein) was detected in the AF of MMC fetuses at E14 (Figure 2B). Both forms of neurocan, representing the full-length neurocan (245 kDa core protein) and the proteolytically cleaved N-terminal neurocan fragment (130 kDa core protein), were present in the AF of MMC fetuses starting at E16 (Figure 2B). Compared with E14, the total level of neurocan in the AF of MMC fetuses was significantly elevated at E16, followed by a smaller but significant increase at E18, reaching the highest level at E21 (Figure 2C). In sharp contrast to these results, the signal for neurocan was not detectable in the AF of normal age-matched fetuses (Figure 2B). Immunoblotting with the 650.24 neurocan antibody, which recognizes the C-terminal epitope (Figure 2A), revealed detection of full-length neurocan, as well as the proteolytically cleaved C-terminal neurocan fragment (150 kDa core protein), in the AF of MMC fetuses starting at E14, with a rapid increase in the intensity of the neurocan signal between E14 and E16 and its peak at E21 (Figure 2B). Only faintly stained bands of neurocan, predominantly corresponding to C-terminal neurocan fragments, were identified in the AF of normal fetuses (Figure 2B). Quantitative analysis showed that the total AF level of neurocan was significantly elevated in MMC compared to normal controls at each examined gestational age (Figure 2D). Moreover, the total level of neurocan in the AF of MMC fetuses robustly increased with gestational time, reaching the highest level at E21 (Figure 2D). Western blotting analyses demonstrated the presence of full-length neurocan, the proteolytically cleaved N-terminal neurocan fragment, and the C-terminal neurocan fragment in MMC spinal cord lysates that were used as positive controls (Figure 2B), indicating that the molecular forms of neurocan identified in the AF of MMC fetuses and MMC spinal cords were qualitatively similar. 

Phosphacan is a secreted spliced extracellular variant of the protein tyrosine phosphatase receptor type Z (PTPRZ; also known as RPTPβ) that is expressed during CNS development and in pathological processes such as tissue injury [19,24]. Western blotting analysis with the 3F8 antibody, which recognizes phosphacan and long receptor type PTPZ (Figure 2A), revealed an approximately 400 kDa single band of core protein, indicating the presence of secreted phosphacan in the AF of MMC fetuses starting at E14 (Figure 2B). In contrast to the AF of MMC fetuses, only weakly stained bands of phosphacan were visible in the AF of normal fetuses at E14, and their intensity diminished to a barely detectable level after E16 (Figure 2B). The total AF levels of phosphacan were significantly higher in MMC than in normal controls at all examined time points and progressively increased in MMC compared to normal age-matched controls as pregnancy advanced (Figure 2E). 

The analyses aimed to evaluate the presence of neurocan and phosphacan were initially conducted using AF samples pooled from three randomly selected MMC or normal fetuses. Typically, RA induces MMC in the majority of fetuses, with the other littermates appearing as normal [20]. To verify that these CSPGs originated from the MMC defect and were not generated by RA exposure, AF samples from individual E21 fetuses with MMC defects, littermates with no MMC defects after RA exposure, and normal controls were loaded into separate lanes and subjected to Western blotting. In contrast to the AF from fetuses with MMC defects, the AF samples from RA-exposed littermates showing no signs of MMC were similar to normal controls containing little or no neurocan and phosphacan (Figure 2F), verifying a direct correlation with the presence of MMC defect.

### 3.2. Secretion of Neurocan and Phosphacan from the MMC Spinal Cord

**Figure 3:**
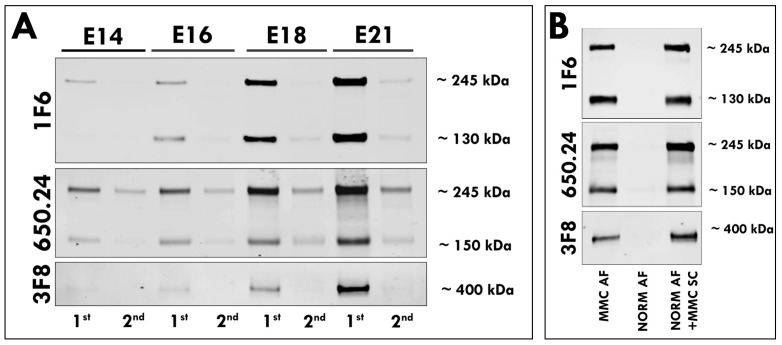
(**A**) Spinal cords isolated from MMC fetuses at E14, E16, E18, and E21 were sequentially extracted with detergent-free buffer (first extract), followed by detergent-containing buffer (second extract), and equal amounts of extracted total protein were digested with ChABC and subjected to Western blotting analysis with 1F6, 650.24, or 3F8 antibodies. Representative Western blot demonstrates that the vast majority of neurocan and phosphacan was detected in the first extract compared with the second extract at all gestational ages. (**B**) Equal volumes of AF samples from E21 MMC fetuses (MMC AF), AF samples from normal age-matched controls (NORM AF), and AF samples following incubation of spinal cords isolated from E21 MMC fetuses in normal age-matched AF (NORM AF + MMC SC) were digested with ChABC and subjected to Western blotting analysis with 1F6, 650.24, or 3F8 antibodies. Western blot demonstrates the presence of the full-length and proteolytically cleaved N-terminal and C-terminal fragments of neurocan and phosphacan in the AF samples following incubation of spinal cords isolated from MMC fetuses in normal AF, which does not contain these proteins. Data represent spinal cord extracts from at least two independent sets of spinal cord tissue samples per age group.

Next, we sought to determine if neurocan or phosphacan from the spinal cord tissue were readily soluble in an aqueous environment. We performed sequential extractions of MMC spinal cord proteins at various points of gestation using detergent-free, followed by detergent-containing, buffer. Immunoblotting of extracted proteins in all examined samples showed that the vast majority of neurocan and phosphacan was present in the first fraction extracted without detergent, with only a minor component identified within the second fraction extracted with detergent (Figure 3A). To further evaluate the release of neurocan and phosphacan into the AF, MMC spinal cord tissue from E21 fetuses was placed into AF samples of aged-matched normal fetuses that did not contain these CSPGs. Western blotting analysis revealed the presence of full-length neurocan and the proteolytically cleaved N-terminal neurocan fragment and C-terminal neurocan fragment, as well as phosphacan, detected in all examined samples of AF incubated with MMC spinal cords (Figure 3B), supporting their release into the AF. 

### 3.3. Expression of Neurocan and Phosphacan in the Developing MMC Spinal Cord 

**Figure 4:**
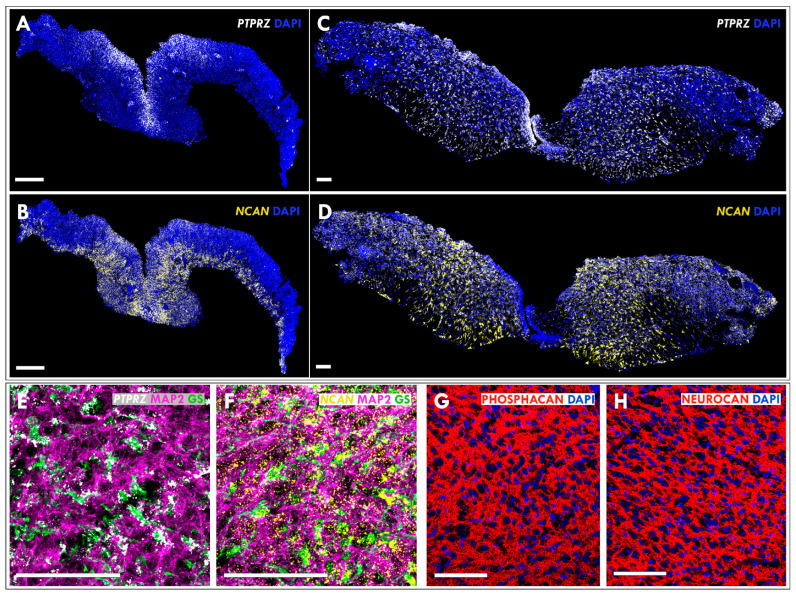
(**A**–**D**) Using RNAscope technology localization of *PTPRZ/Phosphacan* (white) and *Neurocan* (yellow), expression was demonstrated in spinal cord sections from MMC defects examined at E14 (**A**,**B**) and E21 (**C**,**D**). Cell nuclei were fluorescently stained with DAPI (blue). (**E**,**F**) The type of cells that synthetize *RTPRZ/Phosphacan* and *Neurocan* were identified by coimmunostaining for glutamine synthetase (GS; green), a marker of astrocytic cells; MAP2 (magenta), a marker of neurons; and RNAscope for *PTPRZ/Phosphacan* (white; (**E**)); or *Neurocan* (yellow; (**F**)) in spinal cord sections from MMC defects examined at E21. (**G**,**H**) The extracellular expression pattern of neurocan and phosphacan proteins was demonstrated by immunostaining of spinal cord sections from the MMC defect examined at E21. Neurocan and phosphacan immunostainings (red) are shown in combination with DAPI (blue). Scale bar indicates 100 µm. Data represent at least three sections from three fetuses per age group.

Finally, RNAscope analysis in MMC spinal cord sections using specific probes for the *Neurocan* and the *PTPRZ/Phosphacan* revealed enriched *PTPRZ/Phosphacan* expression in the externally exposed ventricular zone, while the site of neurocan expression was extended to the parenchyma of malformed E14 MMC spinal cords (Figure 4A,B). In E21 MMC spinal cords, *Neurocan* and *PTPRZ/Phosphacan* were expressed robustly throughout the spinal cord tissue, with *PTPRZ/Phosphacan* expression maintained in the ventricular zone region (Figure 4C,D). To explore the phenotype of spinal cord cells expressing these CSPGs, RNAscope analysis was combined with coimmunostaining for glutamine synthetase (GS), a marker of astrocytic cells [25] and MAP2, a marker of neuronal cells [26]. Analysis of spinal cord sections from MMC defects demonstrated that the *PTPRZ/Phosphacan* was expressed by astrocytes, while *Neurocan* was expressed by neurons and astrocytes (Figure 4E,F). Consistent with our biochemical studies of spinal cord tissues, immunohistochemical analysis confirmed extracellular localization of secreted neurocan and phosphacan in spinal cord sections from MMC defects (Figure 4G,H). 

## 4. Discussion

In this study, we identified the presence of two CNS-associated CSPGs—neurocan and phosphacan—in the AF of fetal rats in a clinically relevant model of MMC. These MMC fetuses showed robust and significantly elevated levels of neurocan and phosphacan in the AF compared to normal age-matched controls at different developmental time points starting at E14. Normally appearing littermates from the RA-exposed cohort showed no increase in the AF levels of neurocan or phosphacan, unlike those with MMC defects. In addition, we showed that the full-length neurocan, along with its proteolytically cleaved forms and phosphacan identified in the AF of MMC fetuses, was identical to that found in the MMC spinal cords and could be efficiently released from the spinal cord tissue into the normal AF or detergent-free buffer. These data provide evidence for the assessment of neurocan and phosphacan levels within the AF as promising diagnostic biomarkers of open NTD-affected fetuses, even at early gestational ages.

The differences in AF levels of neurocan and phosphacan have not been previously reported between MMC and normal fetuses, although changes in other AF proteins have been studied in MMC. Observations from previous studies in animal models indicate that the molecular components of AF in MMC fetuses, including neurofilament heavy chain expressed by neurons and glial fibrillary acid protein, an intermediate filament specific to astrocytes, might be considerably different from the AF of normal fetuses [27,28]. These molecules are mostly insoluble cytoskeletal proteins with a structural role in axons and astrocytes and are elevated in the AF of MMC fetuses at later stages of gestation, a period corresponding to spinal cord injury in MMC fetuses [27,28]. Neurocan and phosphacan are CSPGs expressed in the developing CNS that are secreted locally and assembled in the surrounding extracellular space [16,17]. Accordingly, our results show an extracellular pattern of neurocan and phosphacan distribution synthetized by astrocytes and neurons in developing MMC spinal cords. Unlike cytoskeletal proteins, neurocan and phosphacan are highly soluble ECM proteins that transfer efficiently from the MMC spinal cord tissue into the AF or detergent-free buffer and are found at exceptionally high levels in the AF of MMC fetuses, even at very early gestational ages (E14). This is consistent with previous reports demonstrating low-affinity attachment of these CSPGs within the neural ECM and their ease of secretion from the spinal cord tissue using detergent-free buffer [18]. Furthermore, immunostaining of spinal cord sections at the site of MMC defect confirmed extracellular localization of these CSPGs, and Western blotting analysis of MMC spinal cord extracts demonstrated identical molecular weight patterns to those identified in the MMC AF. Normally appearing littermates from the RA-exposed cohort showed no increase in the AF levels of neurocan or phosphacan, unlike those with MMC defects. These results support the hypothesis that neurocan and phosphacan are elevated in the AF of MMC fetuses as a result of their release from the site of the MMC defect and not because of their induction by RA exposure. 

The discovery of significantly elevated levels of neurocan and phosphacan in the AF of MMC fetuses provides novel data demonstrating changes in the composition of AF due to the defect and suggests their potential usefulness in early prenatal diagnosis. Most of the established and potential biomarkers for the diagnosis of pregnancy-related pathologies have been identified by analysis of the AF [29,30]. Currently, prenatal testing for alpha fetoprotein (AFP) and acetylcholinesterase (AChE) in the AF and/or ultrasound identification of the defect are considered diagnostic options of open NTDs, while AFP measurement in the maternal serum is used as a prenatal screening test [14,31]. Although AFP assessment during the early second trimester is a biochemical marker for open NTDs, non-neurological fetal disorders are also associated with elevated AFP levels and cannot be excluded based on this marker [14,32]. Unlike AFP, which is a serum protein in the fetus derived from the yolk sac and fetal liver and is not specific for NTDs [14], neurocan and phosphacan are robustly expressed in the developing CNS [16,17]. Using diagnostic biomarkers specific for neural tissue would allow for distinction from non-neurological fetal disorders, therefore increasing the diagnostic accuracy for NTDs. In addition, AFP tests require specific gestational ages, with the optimal time for AFP testing in the maternal serum lying between 1 and 18 weeks of gestation and that in the AF lying between 13 and 22 weeks of gestation, when serum and AF levels of AFP have been found to be substantially elevated in NTD-affected pregnancies [12,14]. Therefore, the interpretation of AFP levels can be challenging in cases of uncertain gestational age. Furthermore, the narrow range of gestational age at which AFP levels are markedly elevated and several other factors, including maternal diabetes or blood contamination of AF samples, may influence the results [12,14]. In some cases, testing for the presence of AChE in the AF can be indicated as an aid to diagnose open NTDs; however, testing for AChE is a non-quantitative method [33]. As demonstrated in the present study, AF levels of neurocan and phosphacan were significantly elevated in comparison to controls from as early as E14, and their content in the AF of MMC fetuses increased with advancing gestational age but decreased in normal AF. These results show that differences in the AF levels of neurocan and phosphacan can be used to discriminate between normal fetuses and those affected by the open NTD at all examined gestational ages. The prominent differences between MMC and normal fetuses are significantly increased AF levels of neurocan and phosphacan detected in MMC fetuses from as early as E14, with their levels in the AF of MMC fetuses raising rapidly at E16 and reaching the highest levels at E21. Comparison of embryonic development between rats and humans indicates that E14 rat embryos are approximately equivalent to gestational week 7 in humans [34]. In this respect, the identification of significant differences in the AF levels of neurocan and phosphacan between MMC and normal rat fetuses makes these molecules promising early biomarkers for detection of open NTDs. These exploratory biomarkers, however, require validation in human samples, including AF and maternal blood, which are less invasive tissue sources from the diagnostic—and especially the screening and monitoring—perspective.

The strengths of this study include a well-characterized fetal rat model of MMC, which, similarly to human MMC, results from incomplete neural tube closure, pathologically resembles human MMC, and provides an excellent translational model [20]. MMC and control specimens collected at various time points allow for a systemic analysis of a large number of samples and age-matched controls during gestation, which is not possible in human pregnancies. In this study, we isolated AF samples from MMC and age-matched controls and used Western blotting to examine the presence of and changes in the AF levels of neurocan and phosphacan in MMC and normal fetuses at four gestational ages. This method enabled us to identify neurocan and phosphacan in the AF and to characterize relative changes in their levels and molecular forms. While Western blotting is an effective method to identify CSPGs and characterize relative changes in their levels, one limitation of this method is that the actual concentrations of neurocan and phosphacan in the AF cannot be established. In future studies, ELISA assays could overcome these limitations. Recent studies show that antibodies and ELISA assays for human CSPGs are commercially available and can be used to establish the concentration of these proteins in human body fluids and may serve as a useful tool for clinical diagnosis [35]. 

## 5. Conclusions

In this pioneering study, we provided evidence for the elevated levels of two CNS-associated CSPGs—neurocan and phosphacan—in the AF of fetal rats displaying MMC defects. We demonstrated that significant differences in the AF levels of neurocan and phosphacan allow for distinction between MMC and normal controls at all examined gestational ages. These findings indicate that elevated levels of neurocan and phosphacan are effective predictors of open NTDs and may constitute potential biomarkers to facilitate early diagnosis. These biomarkers may have the potential to not only improve early diagnosis but also may be beneficial for the assessment of prenatal closure of open NTDs, where decreased AF levels of neurocan and phosphacan indicate treatment efficacy. Based on the presented results, demonstrating a correlation between increased AF levels of neurocan and phosphacan and the presence of MMC defect in fetal rats, future studies of human pregnancies are required to confirm these associations. The identification of sensitive and specific biomarkers can provide diagnostic information beyond what is offered by existing laboratory tests in the field of early detection and diagnosis of open NTDs. Furthermore, the discovery of CNS-associated proteins such as neurocan and phosphacan in the AF of MMC fetuses provides new information about changes in the composition of AF in in the presence of the defect and may also contribute to a better understanding of the underlying pathophysiology and development of prenatal therapies.

## 6. Patents

B.K., G.M.S. and K.J. are inventors of a patent that covers the diagnostic methods using neurocan and phosphacan that are a subject of this article.

## Data Availability

The data that support the findings of this study are available from the corresponding author upon reasonable request.
